# Comprehensive analysis of the prognosis and immune effect of the oncogenic protein Four Jointed Box 1

**DOI:** 10.3389/fonc.2023.1170482

**Published:** 2023-05-30

**Authors:** Mei Huang, Tian Guo, Yan Meng, Ruling Zhou, Man Xiong, Jian Ding, Yali Zhang, Side Liu, Kangmin Zhuang

**Affiliations:** ^1^ Guangdong Provincial Key Laboratory of Gastroenterology, Department of Gastroenterology, Nanfang Hospital, Southern Medical University, Guangzhou, Guangdong, China; ^2^ Pazhou Lab, Guangzhou, Guangdong, China; ^3^ Department of Gastroenterology, Zhuhai People’s Hospital (Zhuhai Hospital Affiliated With Jinan University), Zhuhai, Guangdong, China

**Keywords:** pan-cancer, FJX1, prognosis, biomarker, immunotherapy

## Abstract

**Background:**

The Four Jointed Box 1 (FJX1) gene has been implicated in the upregulation of various cancers, highlighting its crucial role in oncology and immunity. In order to better understand the biological function of FJX1 and identify new immunotherapy targets for cancer, we conducted a comprehensive analysis of this gene.

**Methods:**

We analyzed the expression profiles and prognostic value of FJX1 using data from The Cancer Genome Atlas (TCGA) and Genotype-Tissue Expression (GTEx). Copy number alterations (CNAs), mutations, and DNA methylation were analyzed through cBioPortal. The Immune Cell Abundance Identifier (ImmuCellAI) was used to examine the correlation between FJX1 expression and immune cell infiltration. The relationship between FJX1 expression and immune-related genes and immunosuppressive pathway-related genes was analyzed using The Tumor Immune Estimation Resource version 2 (TIMER2). Tumor mutational burden (TMB) and microsatellite instability (MSI) were obtained from TCGA pan-cancer data. The effect of immunotherapy and the IC50 were assessed using IMvigor210CoreBiologies and Genomics For Drug Sensitivity in Cancer (GDSC). Finally, we evaluated the impact of FJX1 on colon cancer cell proliferation and migration through *in vitro* functional experiments.

**Results:**

Our study indicated that FJX1 expression was high in most cancers and was significantly associated with poor prognosis. High FJX1 expression was also linked to significant alterations in CNA, DNA methylation, TMB, and MSI. Positive correlations were found between FJX1 expression and tumor-associated macrophages (TAMs) and with immune-related genes such as TGFB1 and IL-10 and immunosuppressive pathway-related genes such as TGFB1 and WNT1. On the other hand, FJX1 expression showed a negative relationship with CD8+ T cells. Furthermore, high FJX1 expression led to reduced effectiveness of immunotherapy and drug resistance. In colon cancer cells, FJX1 knockdown was found to decrease cell proliferation and migration.

**Conclusion:**

Our research findings demonstrate that FJX1 is a new prognostic factor with a significant role in tumor immunity. Our results highlight the importance of further exploring the potential of targeting FJX1 as a therapeutic strategy in cancer.

## Introduction

Cancer is currently the leading cause of premature death and reduces life expectancy worldwide (1–3). Although traditional treatments have been developed, some patients may become resistant to them (4, 5). Immunotherapy is a promising treatment that can overcome drug resistance and target escape. With the help of public databases, researchers can identify novel immunotherapy targets and therapeutic strategies through pan-cancer analysis of gene expression (6–8).

One potential target for immunotherapy is four jointed box 1 (FJX1), which is closely related to various tumor pathways in other species (9–13). While its biological function and tumor pathogenesis in human cancer are not fully understood, studies have found that FJX1 is highly expressed in several types of cancer, including head and neck cancer, colon cancer, breast cancer, ovarian cancer, and lung cancer (14–18). Additionally, high FJX1 expression has been linked to poor survival in colon cancer and can regulate important proteins in cell cycle progression to enhance proliferation and invasion in nasopharyngeal carcinoma (19–21). Interestingly, a recent study found that FJX1-specific peptides can inhibit the proliferation of high FJX1 expression cancer cells and may serve as a potential immunotherapy for NPC patients (22). These findings suggest that FJX1 may be a candidate diagnostic and prognostic biological target and an immunotherapy target for cancers. Further research in this area may lead to the development of more effective treatments for cancer patients.

In this study, we conducted a comprehensive analysis of the relationship between FJX1 expression and various types of cancer using pan-cancer data from The Cancer Genome Atlas (TCGA) and Genotype-Tissue Expression (GTEx) databases. We also analyzed copy number alteration, mutation status, and DNA methylation of FJX1 using cBioPortal. In addition, we used Immune Cell Abundance Identifier (ImmuCellAI) to examine the correlation between FJX1 expression and immune cell infiltration.

Furthermore, we investigated the association between FJX1 expression and immune-related genes and immunosuppressive pathway-related genes using The Tumor Immune Estimation Resource version2 (TIMER2). We also assessed the tumor mutational burden (TMB) and microsatellite instability (MSI) using TCGA pan-cancer data. Additionally, we examined the immunotherapy effect and IC50 using IMvigor210CoreBiologies and Genomics For Drug Sensitivity in Cancer (GDSC). To validate our findings, we performed functional experiments *in vitro* to determine whether FJX1 promotes colon cancer cell proliferation and migration. We also co-cultured THP1 macrophages with HCT116-siFJX1. Our results indicated that FJX1 is a critical prognostic factor in various cancers and plays a crucial role in tumor immunity. We believe that the pan-cancer analysis of FJX1 can provide new insights into the development of novel therapeutic strategies for cancer treatment.

## Materials and methods

### FJX1 gene expression analysis

The “ggplot2” R package was used to investigate the FJX1 abnormal expression between 31 types of normal tissue and 33 types of cancer by GTEx (https://commonfund.nih.gov/GTEx) (23) and TCGA (https://portal.gdc.cancer.gov/). We conducted box plots to show the different FJX1 expression between cancerous tissues and paracancerous tissues and in different stages of pathology in numerous tumors, via “ggpubr” and “ggplot” R package, respectively. All the data of TCGA and GTEx for FJX1 were obtained from the UCSC XENA (https://xenabrowser.net/).

### Analysis of genetic variation and gene set variation

Genetic variation characteristics of FJX1 were acquired via cBioPortal (https://www.cbioportal.org/) (24), including mutation type, structural variant, and CNA and DNA methylation. Meanwhile, the CNA and DNA methylation correlation with FJX1 mRNA expression were analyzed by the “ggplot2” R package. We explored the correlation between FJX1 and 50 star pathways in HALLMARK via “GSVA score” R package, and a heat map was made via the “ggplot2” R package.

### Survival prognosis analysis

The FJX1 expression correlation with prognosis for patients were studied via overall survival (OS), disease-free interval (DFI), disease-specific survival (DSS), and progression-free interval (PFI). The HR and p-value were displayed via forest diagram. The FJX1 expression correlation with cancer survival were employed via Kaplan–Meier analysis, and the survival curves were manufactured by “survminer” and “survival” R packages.

### Immune infiltration and immune modulator genes analysis

We used related metrics including immune score, stromal score, ESTIMATE sore, tumor purity, immune-related pathways, metastasis-related pathways, and DNA damage repair-related pathways to explore the FJX1 expression relation with tumor microenvironment in pan-cancer. Meanwhile, we analyzed the FJX1 expression correlation with immune infiltrating cells in various tumors via ImmuCellAI (http://bioinfo.life.hust.edu.cn/ImmuCellAI#!/) (25). Additionally, we used TIMER2 (http://timer.comp-genomics.org/) (26) to explore the FJX1 expression connection with TMB, MSI, immune-suppressive pathway-related genes, and immune-related genes. The results were all displayed by heat maps made by the “ggplot2” R package.

### Immunotherapy analysis

The immunotherapy datasets were obtained from IMvigor210CoreBiologies (http://research-pub.gene.com/IMvigor210CoreBiologies/packageVersions/) to analyze the FJX1 expression relationship with immunotherapy efficacy and overall survival of patients.

### Connection between FJX1 and IC50

The connections between FJX1 expression and IC50 of 198 types of drug were analyzed by using the data from GDSC (https://www.cancerrxgene.org/). The first six drugs with positive correlation were selected and used the “ggplot2” R package to make line chart.

### Cell culture and treatment

Colon cancer cells from human (HCT116 and SW480) and THP1 were obtained from American Type Culture Collection (ATCC, Manassas, VA, USA). HCT116 and SW480 were cultivated in DMEM (Gbico & Trade,China), and THP1 were cultivated in RPMI-1640 (Gbico & Trade, China). We added 10% fetal bovine serum (FBS, ExCell Bio) in media to feed the cells and incubated the cells in an incubator containing 5% CO_2_ at 37°C. For transient transfection, colon cancer cells were transfected with FJX1-siRNA and FJX1-NCRNA using Lipo8000 (Beyotime, Shanghai, China) and DMEM (Gbico & Trade, China), following the manufacturer’s instructions. After 48 h, the real-time quantitative PCR (q-PCR) and Western blot (WB) were used to verify transfection efficiency (FJX1-siRNA 5′-GCACUGUAAGG CCAAGUACTT-3′; FJX1-NCRNA 5′-TTCTCCGAACGTGTCACGT-3). For co-culture, we cultivated THP-1 (5×10^5^) in a 12-well plate and added 200 ng/ml phorbol-12-myristate-13-acetate (PMA) (MedChemExpress, NJ, USA) for 24 h to differentiate into adhered macrophages and used an inverted microscope to record macrophages morphology. Pretreated colon cancer cells (2×10^5^) were seeded in a chamber (0.4 μm pore, Corning, USA), then transferred to the 12-well plate planted with adhered macrophages, and recorded macrophages morphology again after co-culturing for another 24 h. CD80, CD86, and CD163 expressed on co-cultured macrophages were detected by qPCR.

### Cell proliferation assay

The pretreated colon cancer cells were planted into a 96-well plate (1 × 10^3^ cells/well). CCK-8 reagent (Yeasen Bio, shanghai, China) was co-incubated with the cells after 24, 48, 72, 96, and 120 h, respectively, according to the manufacturer’s instruction. OD450 values were determined via a microplate reader.

### Transwell migration assay

We prepared the pretreated colon cancer cells. Complete medium (600 μl) was added in the bottom of a 24-well plate; meanwhile, transwell chambers (0.8 μm pore, Corning, USA) were put in the 24-well plate. A total of 200 μl cell suspension (5×10^4^ cells/well) with serum-free medium was planted in transwell chambers. After incubation for 48 h, we used 4% paraformaldehyde to immobilize the cells and 0.1% crystal violet solution for dyeing, then seriously removed the cells in the upper membrane of the chamber with cotton swabs. An upright microscope was used to photograph, and Image J was used to dealt with the results.

### Wound healing assay

The pretreated colon cancer cells (1 × 10^5^ cells/well) were seeded into 12-well plate until the cells reached 95% confluence. We used a pipette tip to gain a cross scratch and washed the cells three times with phosphate-buffered saline (PBS). Serum medium (3%) was utilized to cultivate the cells, and the inverted microscope was applied to photograph at 0 and 48 h. The scratch areas were assessed via ImageJ.

### Real-time quantitative PCR

Total RNA of colon cancer cells and macrophages was extracted by the TRIzol reagent (Leagene, Beijing, China), and EVO M-MLV RT Premix (Accurate Bio, Hunan, China) was used to perform reverse transcription to obtain objective cDNA. FJX1, TGB1, IL10, CD80, CD86, and CD163 expressions were detected by SYBR Green PCR Master Mix (GenStar, Beijing, China). GAPDH was a control reference, and the classical 2^−ΔΔCt^ method was applied to calculate the relative expression. Primers are detailed in the attachment.

### Western blot analysis

FJX1 proteins were extracted from the cells through standard protocols, separated by sodium dodecyl sulfate (SDS)-polyacrylamide gel electrophoresis, and performed Western blot analyses. The chemi-luminescence method was used to detect protein bands. Primary antibody against FJX1 (1:1,000, ABclconal, Wuhan, China) was used. GAPDH (1:10,000, ABclconal, Wuhan, China) was used as a control. The secondary antibodies were anti-rabbit (1:10,000, ABclconal, Wuhan, China) and anti-rat (1:10,000, ABclconal, Wuhan, China).

### Statistical analysis

The correlation coefficients are all Pearson, but the Spearman coefficient is used in the correlation analysis of IC50. All experimental data analysis and picture production were done through GraphPad Prism 9.0. Statistical analyses were performed with Student’s t-test. Each experiment was repeated three times. All p < 0.05 was considered statistically significant.

## Results

### FJX1 expression status analysis in pan-cancer

**Figure 1:**
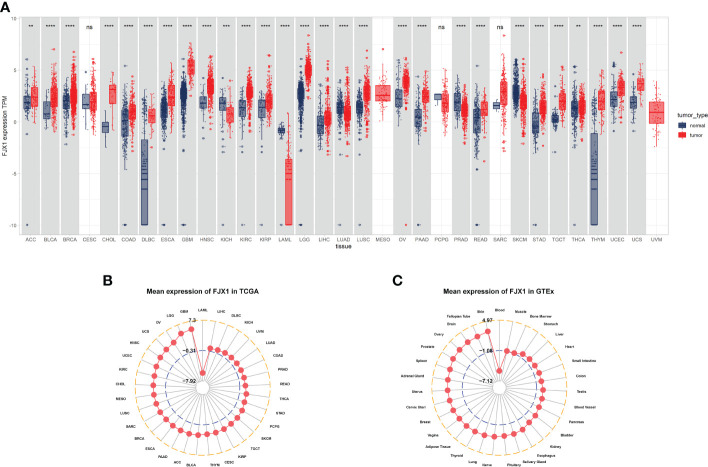
FJX1 expression levels vary in different cancers. **(A)** Profiles of FJX1 levels between tumors and normal tissues. **(B)** Expression of FJX1 in 33 types of cancer (TCGA). **(C)** Expression of FJX1 in 31 types of normal tissue (GTEx). The box plots and radar charts were made by “ggplot2” and “ggradar” R package, respectively. *p < 0.05, **p < 0.01, ***p < 0.001, ****p < 0.0001. ns, not significant.

The FJX1 expression of cancer tissues correlation with normal tissues were explored by TCGA and GTEx. The FJX1 expression in cancer tissues was significantly higher than in normal tissues, including the adrenocortical carcinoma (ACC), bladder urothelial carcinoma (BLCA), breast invasive carcinoma (BRCA), cholangiocarcinoma (CHOL), COAD, lymphoid neoplasm diffuse large B-cell lymphoma (DLBC), esophageal carcinoma (ESCA), glioblastoma (GBM), head and neck squamous cell carcinoma (HNSC), brain lower grade glioma (LGG), kidney renal clear cell carcinoma (KIRC), kidney renal papillary cell carcinoma (KIRP), liver hepatocellular carcinoma (LIHC), lung squamous cell carcinoma (LUSC), ovarian serous cystadenocarcinoma (OV), pancreatic adenocarcinoma (PAAD), rectum adenocarcinoma (READ), stomach adenocarcinoma (STAD), thyroid carcinoma (THCA), testicular germ cell tumors (TGCT), thymoma (THYM), uterine corpus endometrial carcinoma (UCEC), and uterine carcinosarcoma (UCS). On the contrary, the FJX1 expression in cancer tissues was lower significantly, compared with normal tissues, including the kidney chromophobe (KICH), acute myeloid leukemia (LAML), lung adenocarcinoma (LUAD), prostate adenocarcinoma (PRAD), and skin cutaneous melanoma (SKCM) (**Figure 1A**). Simultaneously, the radar charts displayed that the mean FJX1 expression in cancers was 7.3, while the mean FJX1 expression in normal tissues was 4.97 (**Figures 1B, C**). Additionally, we analyzed the FJX1 expression in cancer and para-cancerous tissues. In BLCA, BRCA, CHOL, COAD, ESCA, HNSC,SARC, KIRC, KIRP, LIHC, STAD, and THCA, the FJX1 expression in cancer was significantly higher than in paracancerous tissues. Inversely, FJX1 expression in cancer was lower than in paracancerous tissues only in KICH (**Supplementary Figure S1A**). We also investigated the FJX1 expression levels in different clinical stages. The FJX1 expression increased with tumor stage in ACC, COAD, ESCA, KIRP, LUAD, and UVM (**Supplementary Figure S1B**). All the investigations indicated that FJX1 expression was significantly upregulated in most cancers and associated with tumor stage.

### FJX1 CNA and DNA methylation analysis in pan-cancer

**Figure 2:**
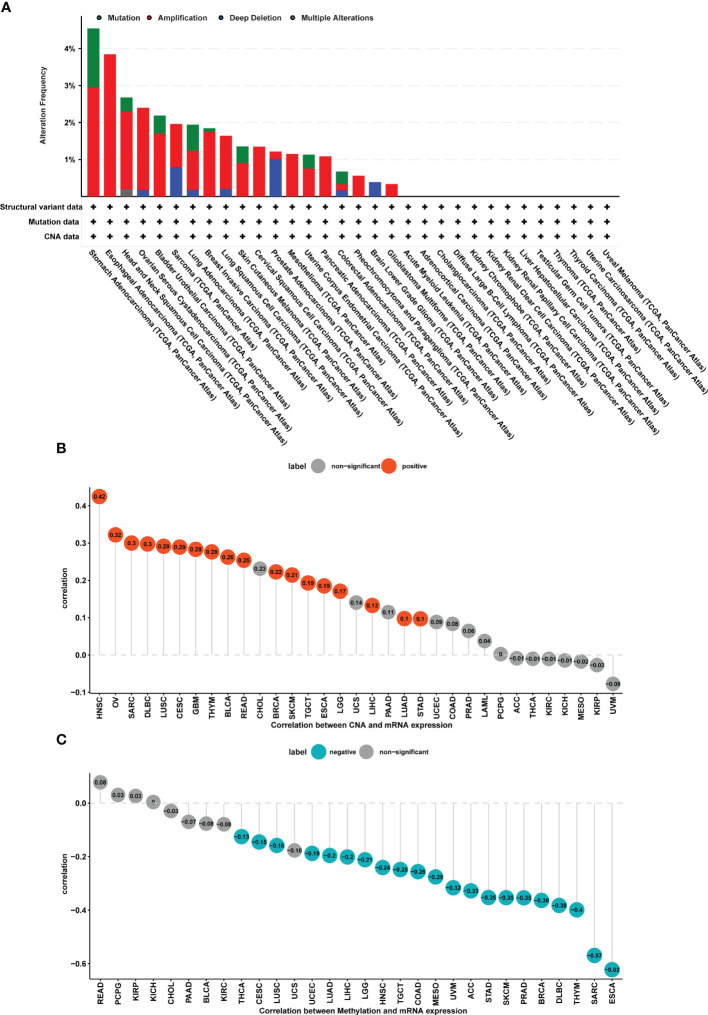
CNA and DNA methylation of FJX1 in pan-cancer. **(A)** The structural variant, mutation, and CNA status of FJX1 in TCGA tumors (cBioportal). **(B)** Correlation between CNA and FJX1 mRNA expression. Red color represents significant results (p < 0.05). **(C)** Correlation between DNA methylation and FJX1 mRNA expression. Blue color represents significant results (p < 0.05). CNA, copy number alteration.

The FJX1 gene alterations data were obtained from the cBioPortal, which suggested that the highest alteration frequency of FJX1 was more than 4% and the “amplification” was the primary genetic alteration type in stomach adenocarcinoma. Among the different types of genetic, variation, “mutation” had the highest expression in stomach adenocarcinoma, “amplification” had the highest expression in esophageal adenocarcinoma, and “deep deletion” had the highest expression in prostate adenocarcinoma (**Figure 2A**). Additionally, we also explored the correlation of FJX1 mRNA expression with CNA and DNA methylation. CNA and FJX1 mRNA expressions were positively correlated in 17 types of cancer, including HNSC, OV, SARC, DLBC, LUSC, GBM, THYM, BLCA, READ, BRCA, SKCM, TGCT, ESCA, LGG, LIHC, LUAD, and STAD (**Figure 2B**), Meanwhile, the DNA methylation and FJX1 mRNA expression were negatively correlated in 21 types of cancers, including THCA, CESC, LUSC, UCEC, LUAD, LIHC, LGG, HNSC, TGCT, COAD, MESD, UVM, ACC, STAD, SKCM, PRAD, BRCA, DLBC, THYM, SARC, and ESCA (**Figure 2C**).

### FJX1 prognostic value analysis in pan-cancer

**Figure 3:**
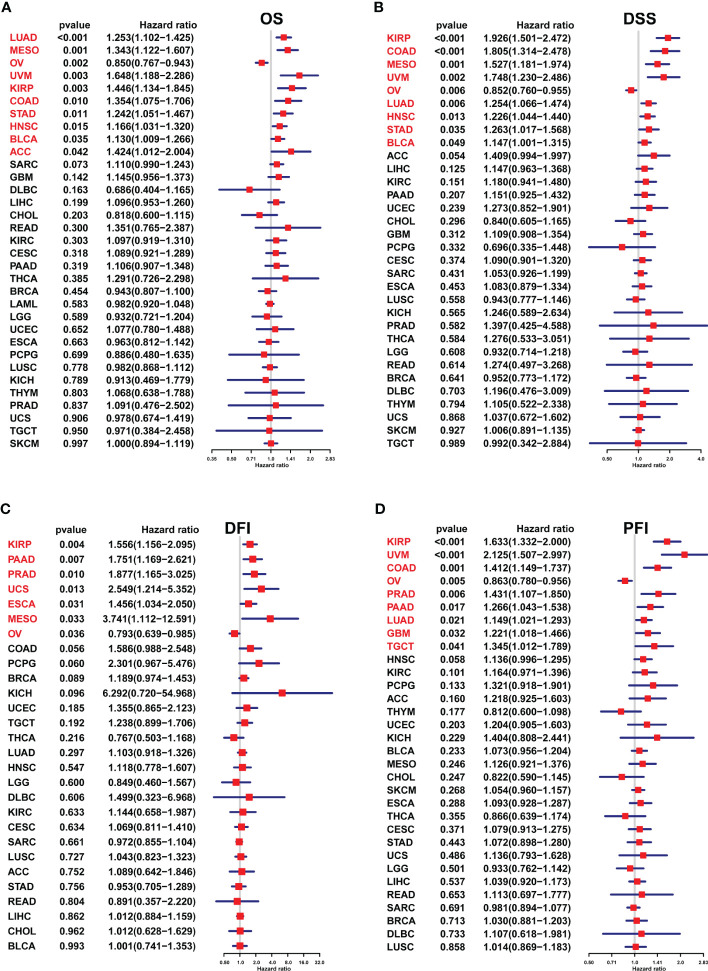
Association between the FJX1 expression and prognostic value in pan-cancer. **(A–D)** The forest plots showing the correlation between FJX1 expression and OS, DSS, DFI, and PFI in TCGA cancers. Red color represents significant results (p < 0.05).

A univariate Cox regression model was employed to analyze the FJX1 expression correlation with OS, DSS, DFI, and PFI in multiple cancers. For OS, high FJX1 expression was significantly linked to worse OS in LUAD, MESO, UVM, KIRP, COAD, STAD, HNSC, BLCA, and ACC (**Figure 3A**). For DSS, low FJX1 expression had a high DSS rate in patients with KIRP, COAD, MESO, UVM, LUAD, HNSC, STAD, and BLCA (**Figure 3B**). For DFI, in KIRP, PAAD, PRAD, UCS, ESCA, and MESO, lower DFI was significantly related with high FJX1 expression (**Figure 3C**). For PFI, high FJX1 expression was significantly related to lower PFI in KIRP, UVM, COAD, PRAD, PAAD, LUAD, GBM, and TGCT (**Figure 3D**). However, in OV, low FJX1 expression implied better OS, DFI, PFI, and DSS (p<0.05, **Figure 3**). Moreover, the survival curve displayed that high FJX1 expression indicated worse overall survival time in 16 types of cancer (**Supplementary Figure S2**). All the results displayed that FJX1 was a potential novel prognostic biomarker.

### Gene set variation analysis of FJX1 in pan-cancer

GSVA were used to investigate the FJX1 expression correlation with 50 stars pathways in HALLMARK. we found that FJX1 had a significantly positive correlation with the first six pathways in various cancers, including “ANGIOGENESIS,” “WNT BETA CATENIN SIGNALING,” “NOTCH SIGNALING,” “EPITHELIAL MESENCHYMAL TRANSITION,” “APICAL JUNCTION,” and “TGF BETA SIGNALING,” which all were closely related to carcinoma and immunity (**Supplementary Figure S3**).

### Immune infiltration and immune modulator genes analysis of FJX1 in pan-cancer

Tumor microenvironment (TME) data were downloaded from TIMER2. As displayed in **Supplementary Figure S4A**, FJX1 expression was positive relation with stromal score, ESTIMATE sore, and immune score in 17, 14, and 10 kinds of cancer, respectively (p<0.05), while there was a negative correlation with tumor purity in 13 kinds of cancer (p<0.05). In addition, FJX1 also had significant positive correlation with immune-related pathways and DNA damage repair-related pathways in most cancers (**Supplementary Figure S4B**).

**Figure 4:**
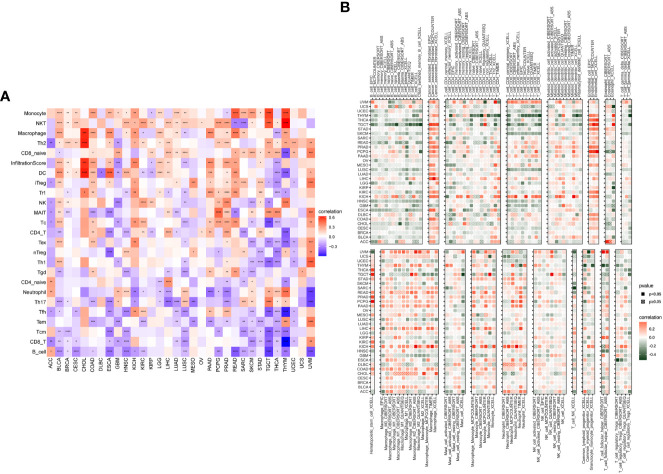
Correlation between FJX1 and immune infiltrating cells in TCGA cancers. **(A)** The correlation between FJX1 expression and immune cells. **(B)** The correlation between FJX1 expression and different immune cell subtypes. Red represents positive correlation, blue or dark green represents negative correlation, and the darker the color, the stronger the correlation.*p < 0.05, **p < 0.01, ***p < 0.001, ****p < 0.0001.

We used ImmuCellAI and TIMER2 to investigate the FJX1 expression relationship with immune infiltrating cells in various TCGA tumors. FJX1 expression was positive relevant with large number of infiltrated immune cells, such as monocyte cells, NKT, macrophages, and Th2, while there was negative association with CD8+ T cells and B cells in various cancers (**Figure 4A**). Additionally, we further evaluated the FJX1 expression relationship with different subtypes of immune cell. We discovered that in most cancers, the FJX1 expression positively related with different subtypes of tumor macrophages (TAMs) but negatively related with different subtypes of B and T cells (**Figure 4B**).

**Figure 5:**
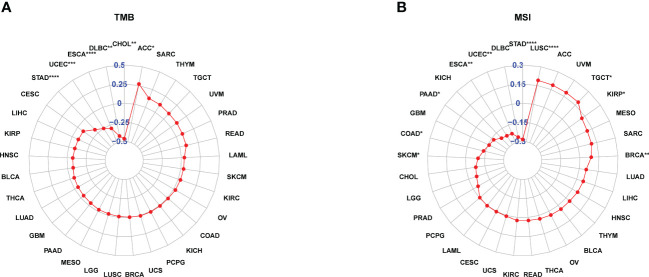
FJX1 correlation with TMB and MSI. **(A)** FJX1 was significantly correlated with TMB in ACC, STAD, UCEC, ESCA, DLBC, and CHOL. **(B)** FJX1 has significantly correlation with MSI in LUSC, TGCT, KIRP, BRCA, SKCM, COAD, PAAD, ESCA, UCEC, and STAD. TMB, mutational burden; MSI, microsatellite instability. *p < 0.05, **p < 0.01, ***p < 0.001, ****p < 0.0001.

**Figure 6:**
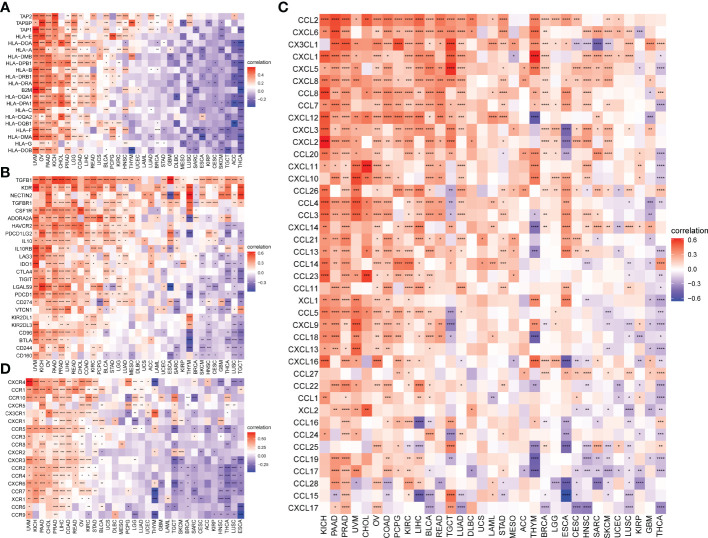
Correlation between FJX1 expression and immune-related genes. Correlation between FJX1 and **(A)** MHC genes, **(B)** Immunosuppressive genes, **(C)** chemokines, and **(D)** chemokine receptors. Red represents positive correlation, blue represents negative correlation, and the darker the color, the stronger the correlation.*p < 0.05,**p < 0.01, ***p < 0.001, ****p < 0.0001.

**Figure 7:**
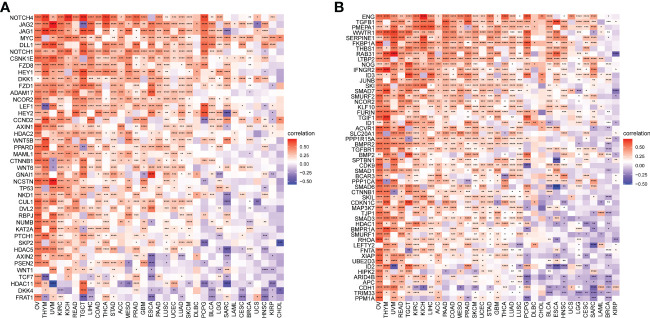
Correlation between FJX1 and immunosuppressive pathways-related genes **(A, B)**. Red represents positive correlation, blue represents negative correlation, and the darker the color, the stronger the correlation.*p < 0.05, **p < 0.01, ***p < 0.001, ****p < 0.0001.

TMB and MSI scores were downloaded to analyze the FJX1 expression relationship with TMB or MSI via TCGA. The results suggested that FJX1 had significant correlation with TMB in ACC, STAD, UCEC, ESCA, DLBC, and CHOL (**Figure 5A**), with MSI in LUSC, TGCT, KIRP, and BRCA, SKCM, COAD, PAAD, ESCA, UCEC, and STAD (**Figure 5B**). Furthermore, we also explored the connection of FJX1 expression with immune-related genes (MHC genes, immunosuppressive genes, chemokines, and chemokines receptors) and immunosuppressive pathway-related genes. We found that FJX1 expression was significantly correlation with vast majority of MHC genes (21 types) in most cancers (**Figure 6A**). Additionally, FJX1 expression was significantly and positively correlated with immunosuppressive genes (TGFB1 and IL-10), chemokines (CCR1 and CCR5), chemokines receptors (CCL2 and CXCL5) (**Figures 6B–D**), and immunosuppressive pathway-related genes (TFGB1 and WNT1), in most TCGA cancers (**Figure 7**). Interestingly, TFGB1 and WNT relative pathway activation was associated with immunosuppressive status. All the investigations revealed that FJX1 was closely relevant to the immunosuppressive microenvironment and the matrix microenvironment. It was indicated that high FJX1 expression put patients in an immunosuppressed state.

### Immunotherapy analysis of FJX1 in pan-cancer

To investigate whether FJX1 affects the immunotherapy effect in cancer patients, we downloaded the immunotherapy dataset from IMvigor210CoreBiologies and found that in the immunotherapy-tolerant group, the FJX1 expression was higher compared with immunotherapy-effective group (p<0.05) (**Supplementary Figure S5A**). In addition, we also found that compared with low FJX1 expression, patients in the high FJX1 expression group had worse overall survival (p=0.00029) (**Supplementary Figure S5B**). Furthermore, stable disease (SD)/progressive disease (PD) accounted for 87% and complete remission (CR)/partial remission(PR) accounted for 13% in patients with high FJX1 expression, while SD/PD accounted for 73%, and CR/PR accounted for 27% in patients with low FJX1 expression (**Supplementary Figure S5C**). All the results suggested that upregulated FJX1 could reduce the efficacy of immunotherapy.

### Connection between FJX1 expression and IC50 in pan-cancer

We obtained the data from GDSC to explain the FJX1 expression connection with IC50 of 198 types of drug. As shown in **Supplementary Figure S6**, FJX1 had significantly positive correlation with IC50 of LGK974, BMS-754807, Crizotinib, AZD5991, Vorinostat, and ML_323, which revealed that patients with high FJX1 expression may develop resistance to these drugs.

### FJX1 knockdown weakens the proliferation and migration in COAD cells

**Figure 8:**
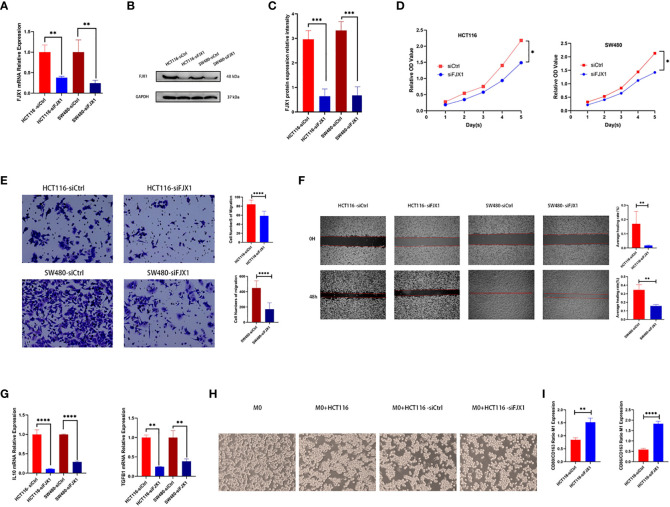
Knocking down FJX1 inhibits proliferation and migration in HCT116 and SW480 cells. **(A)** Real-time PCR and **(B, C)** Weston blot determine the efficiency of knocking down FJX1; **(D)** proliferation assay, **(E)** transwell migration assay, and **(F)** wound healing assay shows that knockdown FJX1 significantly weakens proliferation, migration ability, and average healing rate. The cell numbers of migration and average healing rate(%) are shown in histograms. **(G)** TGFB1 and IL10 mRNA relative expression. **(H)** Morphology of macrophages after co-culture with HCT116 in different states. **(I)** CD80/CD163 and CD86/CD163 are increasing in macrophages after co-culture with HCT116-siFJX1. *p < 0.05, **p < 0.01, ***p < 0.001, ****p < 0.0001. Each experiment was repeated three times. Statistical analyses were performed with Student’s t-test.

The FJX1 was knocked down via transfection with FJX1-siRNA. FJX1 mRNA and FJX1 protein expressions were all lower in the FJX1-siRNA group than in the FJX1-NCRNA group (**Figures 8A–C**). Meanwhile, TGFB1 and IL10 mRNA relative expression were also lower in the FJX1-siRNA group compared with the FJX1-NCRNA group (**Figure 8G**). To confirm the biological function of FJX1 in COAD cells, cell proliferation assay, transwell migration assay, and wound healing assay were performed in HCT116 and SW480 cells. The outcomes indicated that the proliferation and migration ability and wound average healing rate of HCT116 and SW480 cells were attenuated in the FJX1-siRNA group compared with the FJX1-NCRNA group (p all <0.05, **Figures 8D–F**).

### THP1 macrophages co-cultured with knocking down FJX1 HCT116 increased CD80/CD163 and CD86/CD163

As shown in **Figure 8H**, THP1 macrophages were round without antennae. After co-culture with HCT116 bare bead cells and control groups, some THP1 macrophages were elongated and grew antennae, but most of them showed roundness and decreased antennae after co-culture with HCT116-siFJX1. Additionally, according to the results of qPCR, CD80/CD163 and CD86/CD163 were elevated in the knockdown FJX1 group compared with the control group (**Figure 8I**). This suggested that FJX1 had the potential to induce THP1 macrophages to polarize to M2.

## Discussion

Cancer can have a significant impact on a patient’s health and quality of life, causing great suffering. Despite advancements in cancer diagnosis and treatment, the overall survival rate for cancer patients remains unsatisfactory (27). Therefore, it is crucial to explore novel strategies for cancer diagnosis and treatment, and pan-cancer analysis can provide new ideas and directions (28). Previous studies have shown that FJX1 is highly expressed in some cancers (14–18), and in colorectal carcinoma, upregulated FJX1 is significantly associated with poor survival (20). Our findings are consistent with these studies, as our pan-cancer analysis revealed high FJX1 expression in 22 types of cancer, and it was correlated with poor overall survival, disease-specific survival, disease-free interval, progression-free interval, and worse overall survival in some cancers. Therefore, our pan-cancer prognosis value analysis of FJX1 demonstrates that it could be an underlying and novel diagnostic and prognostic biomarker for cancers.

Tumorigenesis is closely associated with various genetic alterations, including mutation, amplification, deep deletion, copy number alteration (CNA), and DNA methylation of genes (29–31). According to our results, FJX1 was found to be altered in 19 types of cancer, with amplification being the most common genetic alteration across different cancer types. Additionally, FJX1 mRNA expression was positively correlated with CNA and negatively correlated with DNA methylation in 18 and 22 types of cancer, respectively. We also discovered that FJX1 is closely linked with cancer and immunity pathways. Previous research has revealed that FJX1 is a direct target of the Hippo-Yes-associated protein in the Hippo-signaling pathway, which regulates cell proliferation and apoptosis (32). Moreover, FJX1 has been shown to promote angiogenesis in colorectal carcinoma and potentiate invasion by regulating planar cell polarity, which is involved in wound repair and development (33). Our external experimental results also demonstrated that knocking down FJX1 in colon cancer cells weakened their proliferation and migration. Thus, all these findings suggest that FJX1 is a factor in promoting carcinogenesis.

The tumor microenvironment (TME) exerts a long-lasting impact on tumor cells and plays critical roles in various aspects of tumor biology, including infiltration, invasion, metastasis, and response to immunotherapy (34). Among the cellular components of TME, macrophages are particularly important and are commonly referred to as tumor-associated macrophages (TAMs) (35). TAMs are a heterogeneous population that can exhibit distinct phenotypes, with the M1 type having an anti-tumor function and the M2 type promoting tumor growth and progression. The proportion of M2 TAMs has been shown to correlate with poor prognosis in many types of cancer (36–38). In our study, we found that the expression of FJX1 was significantly associated with monocytes, macrophages, Th2 cells, and NKT cells. Moreover, we observed a positive and significant correlation between FJX1 expression and most macrophage subtypes. Interestingly, we also found that co-culture of THP1 macrophages with HCT116 cells that were transfected with siFJX1 led to morphological changes in macrophages, with decreased antennae and a more rounded shape. Furthermore, the expression of surface markers such as CD80/CD163 and CD86/CD163 was increased in macrophages co-cultured with HCT116-siFJX1 compared to the control group *in vitro*. CD80 and CD86 are typical markers of M1 macrophages, while CD163 is a marker of M2 macrophages (39). Thus, the increased expression of CD80/CD163 and CD86/CD163 in macrophages suggests a decrease in the proportion of CD163+ M2 TAMs and an increase in the proportion of CD80+ or CD86+ M1 TAMs. Collectively, our findings suggest that FJX1 is positively associated with TAMs.

TMB and MSI are important biomarkers for evaluating antitumor responses and predicting the efficacy of tumor immunotherapy, including antibody therapies and checkpoint inhibitors (40, 41). However, cancer cells can develop drug resistance by undergoing immunoediting, which allows them to escape detection and clearance by the immune system (42, 43). Our research suggests that FJX1 plays a significant role in the development of drug resistance in 6 and 10 types of cancer, by affecting TMB and MSI, respectively. Specifically, high expression of FJX1 is associated with a more immunotherapy-tolerant microenvironment and lower overall survival in cancer patients. Furthermore, our results indicate that FJX1 is positively correlated with the expression of MHC genes, immunosuppressive genes, chemokines, chemokine receptors, and immunosuppressive pathway-related genes in most TCGA cancers. We also found that FJX1 expression is positively associated with TGFB1 and IL-10, which can induce macrophages to M2 polarization and regulate tumor immunology (44). When FJX1 was knocked down in colon cancer cells, the expression of TGFB1 and IL-10 also decreased, suggesting that FJX1 may affect the polarization of macrophages and thus the tumor microenvironment.

Finally, we used GDSC to analyze the connection between FJX1 and IC50 in 198 types of drug and found that high expression of FJX1 is associated with drug resistance. These results suggest that FJX1 is a potential target for the development of immunosuppressants. Overall, our findings provide new insights into the role of FJX1 in cancer immunotherapy and drug resistance.

While our article highlights the significance of FJX1 as a biomarker for carcinogenicity and prognosis in various types of cancer, there are some important limitations to our study. Although previous research suggests that high FJX1 expression is associated with poor prognosis in different tumors, the specific mechanism and role of the tumor immunosuppressive microenvironment have not been fully explored. Therefore, further investigation is necessary to confirm the relationship between FJX1 and the immunosuppressive microenvironment in human cancers. In addition, future studies should also focus on exploring the expression and function of FJX1 in greater detail.

## Conclusion

Our study underscores the importance of FJX1 as a potential biomarker for cancer diagnosis and prognosis. High FJX1 expression may contribute to an immunosuppressive microenvironment, and targeting FJX1 could be a promising approach for immunotherapy in cancer treatment.

## Data availability statement

The original contributions presented in the study are included in the article/**Supplementary Material**. Further inquiries can be directed to the corresponding authors.

## Author contributions

KMZ conceived the study. KMZ ,SDL and YLZ drafted the manuscript and performed the analysis. YM and JD performed the literature search and collected the data. MX contributed to the in-vitro experiment and revision of manuscript. MH contributed to drafting the manuscript and interpreting data. TG and RLZ contribute to the revision of manuscript. MH, TG, YM and RLZ contribute equally to the research. All authors read and approved the final manuscript.
