# Supplementary material for: Comprehensive analysis of the prognosis and immune effect of the oncogenic protein Four Jointed Box 1
Source: Front Oncol. 2023 May 30;13:1170482. doi: 10.3389/fonc.2023.1170482 (PMC10266275; doi:10.3389/fonc.2023.1170482)
Supplement: Supplementary file 1 [file DataSheet_1.docx]

GAPDH forward primer:GGAGTCCACTGGCGTCTTCA,

GAPDH reverse primer:GTCATGAGTCCTTCCACGATACC,

FJX1 forward primer:GCTGGTGGACCTAGTACAATGG,

FJX1 reverse primer:CTTACGAGCCGGTCGAAGTTG,

TGB1 forward primer:CTAATGGTGGAAACCCACAACG,

TGB1 reverse primer:TATCGCCAGGAATTGTTGCTG,

IL10 forward primer:TCAAGGCGCATGTGAACTCC,

IL10 reverse primer:GATGTCAAACTCACTCATGGCT,

CD80 forward primer: AAACTCGCATCTACTGGCAAA,

CD80 reverse primer:GGTTCTTGTACTCGGGCCATA,

CD86 forward primer:CTGCTCATCTATACACGGTTACC,

CD86reverse primer:GGAAACGTCGTACAGTTCTGTG,

CD163 forward primer:GCGGGAGAGTGGAAGTGAAAG,

CD163 reverse primer:GTTACAAATCACAGAGACCGCT.
