# Supplementary material for: Comprehensive analysis of the prognosis and immune effect of the oncogenic protein Four Jointed Box 1
Source: Front Oncol. 2023 May 30;13:1170482. doi: 10.3389/fonc.2023.1170482 (PMC10266275; doi:10.3389/fonc.2023.1170482)
Supplement: Supplementary file 2 [file DataSheet_2.docx]

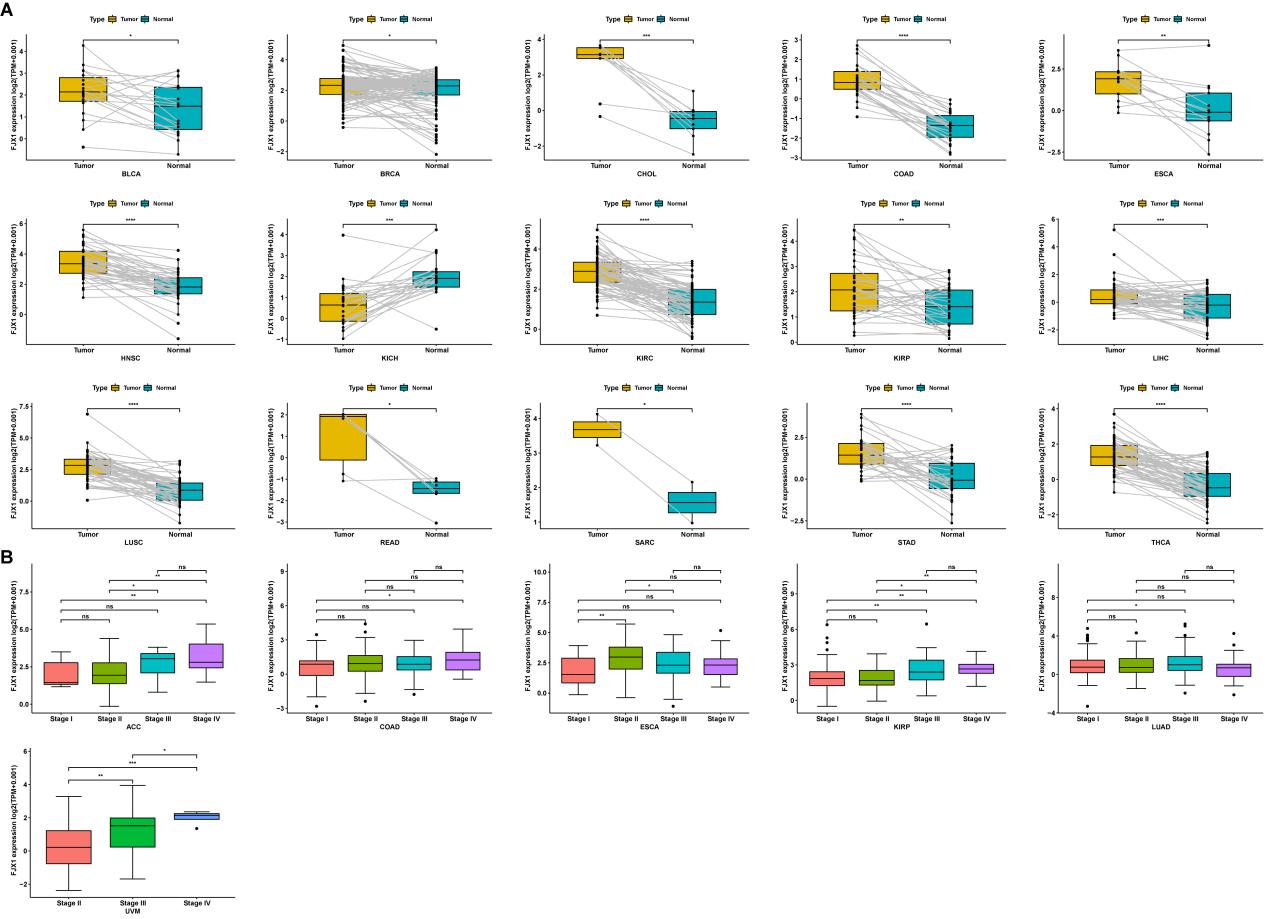


Supplementary figure 1: Patterns of FJX1 expression levels between paired tumors and adjacent tissues and in different pathological stages of cancers. (A) Expression levels of FJX1 between paired tumors and adjacent tissues in BLCA, BRCA, CHOL, COAD, ESCA, HNSC, KIRC, KIRP, LIHC, SARC, STAD and THCA. (B) FJX1 expression in different pathological stages in ACC, COAD, ESCA, KIRP, LUAD and UVM. *P < 0.05, **P < 0.01, ***P < 0.001.


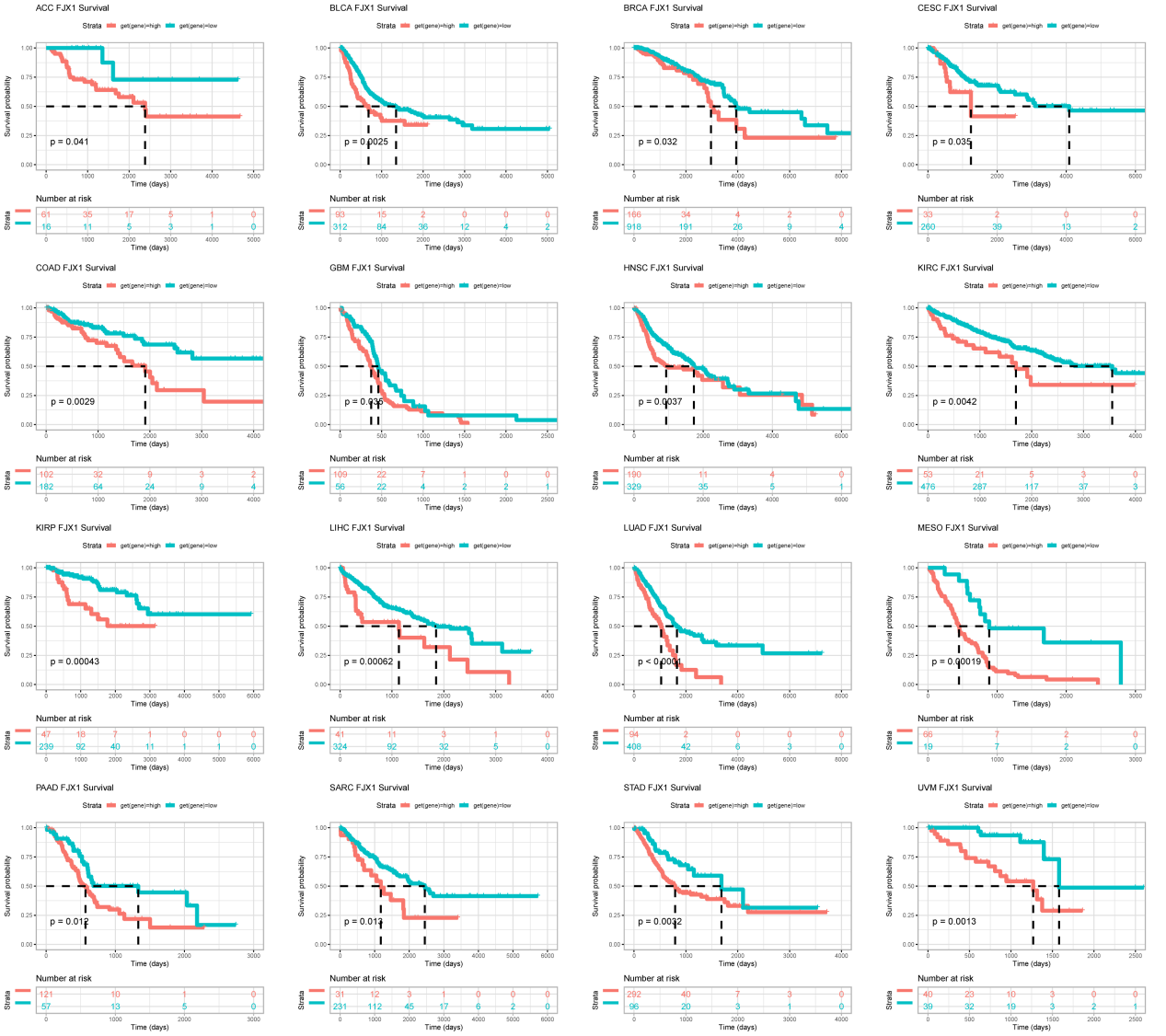
Supplementary figure 2: Kaplan-Meier curves displaying higher expression of FJX1 was related to worse OS in ACC, BLAC, DRCA, CESC,COAD, GBM, HNSC, KIRC, KIRP, HILC, LUAD, MESO, PAAD, SARC, STAD and UVM. The survival curves with log-rank p<0.05 are given.


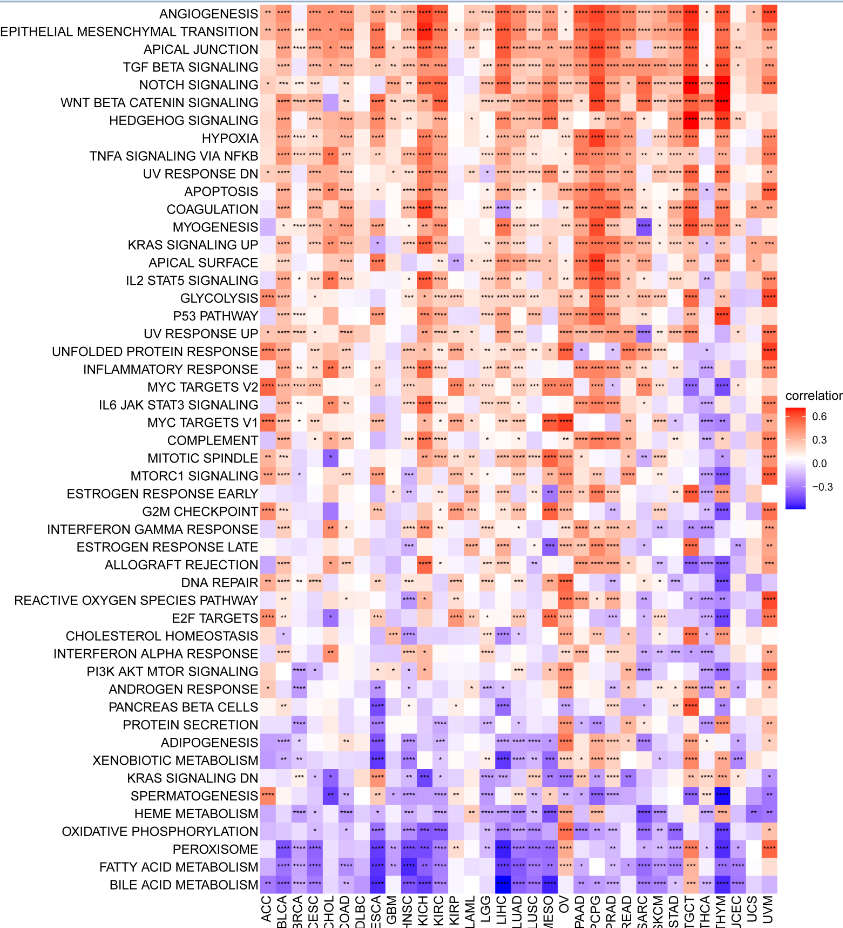


Supplementary figure 3: Correlation between FJX1 and 50 star pathways in HALLMARK. Red represents positive correlation, blue represents negative correlation, and the darker the color, the stronger the correlation. *P < 0.05, **P < 0.01, ***P < 0.001, ****P < 0.0001.


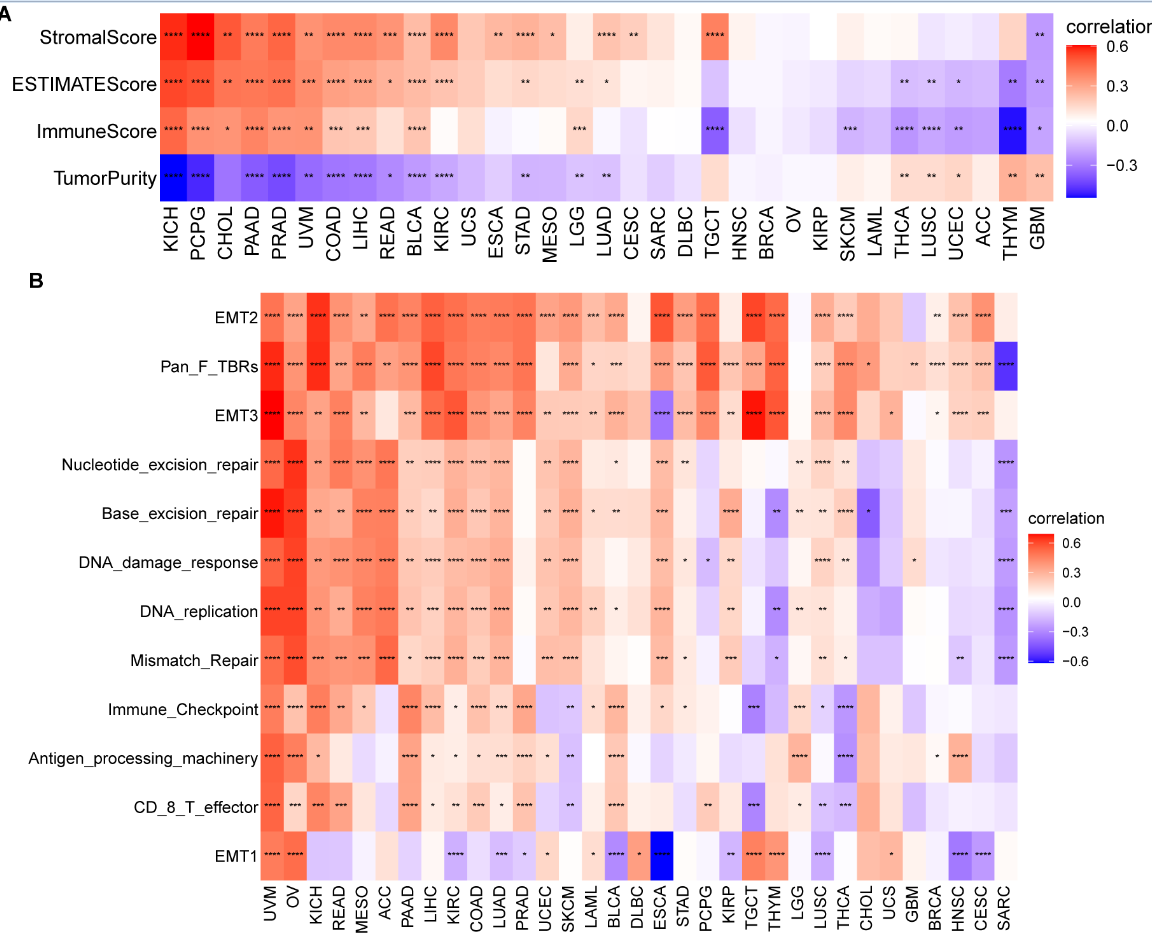


Supplementary figure 4: Correlation between FJX1 and TME. (A) The correlation beetween FJX1 and stromal score, ESTIMATE sore, immune score and tumor purity in TCGA cancers. (B)The correlation between FJX1 and immune-related pathways , DNA damage repair-related pathways and metastasis-related pathways in TCGA cancers. Red represents positive correlation, blue represents negative correlation, and the darker the color, the stronger the correlation.*P < 0.05, **P < 0.01, ***P < 0.001, ****P < 0.0001.TME: tumor microenvironment


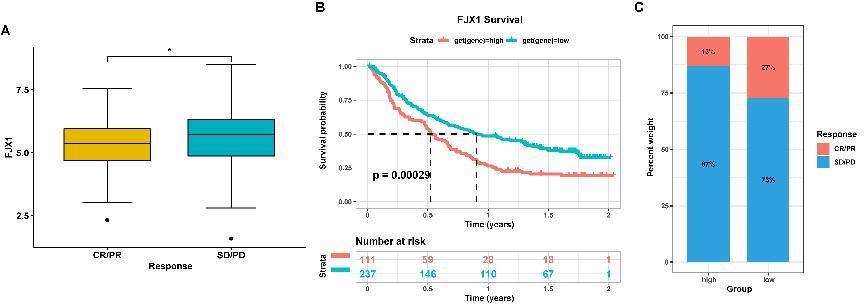


Supplementary figure 5: FJX1 affects the efficacy of immunotherapy. The higher FJX1 expression, (A) the worse response to efficacy, (B)the lower overall survival in patients and (C) the higher SD/PD. CR：complete remission, PR：partial remission, SD: stable disease, PD: progressive disease. *P < 0.05.


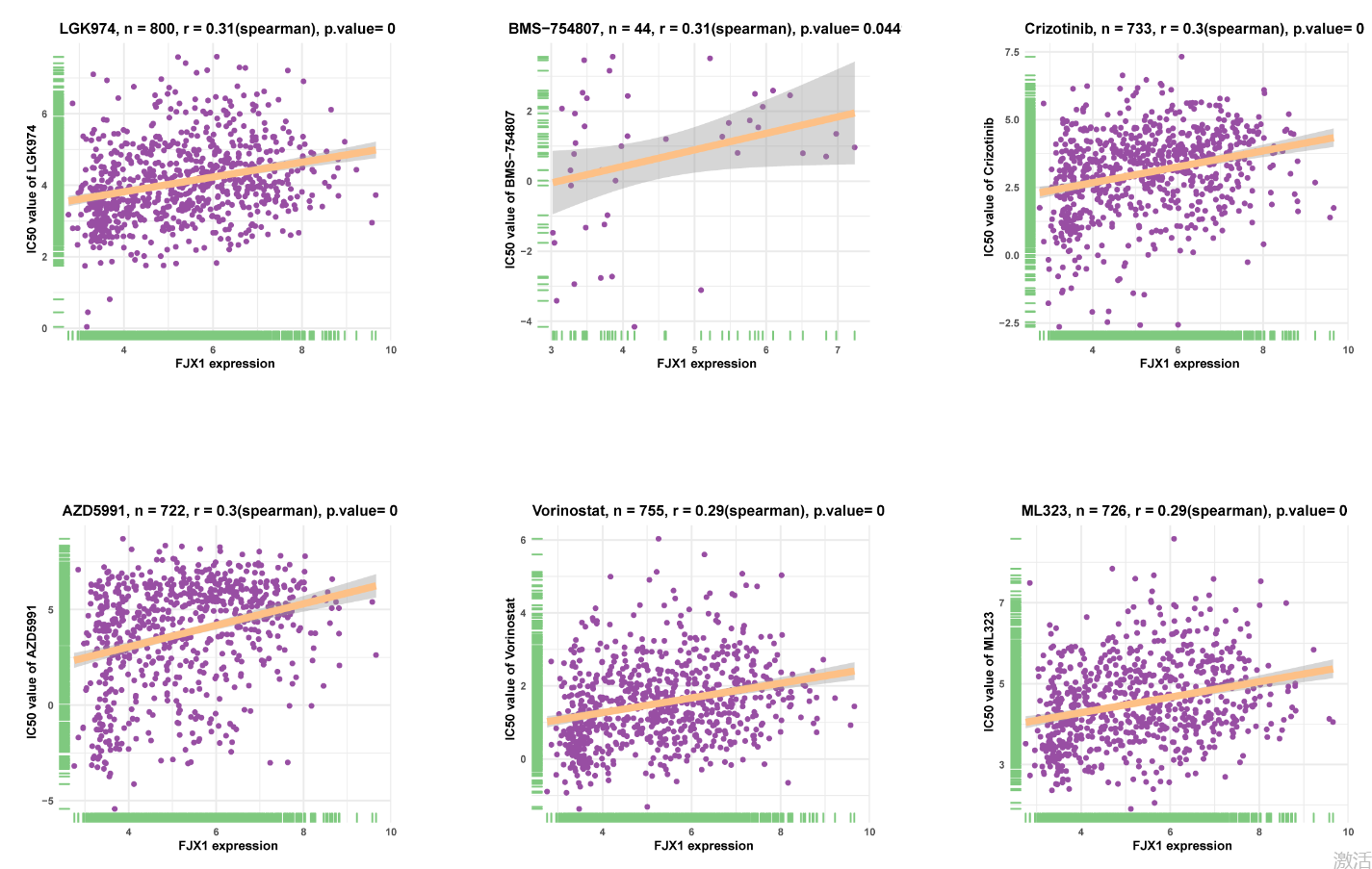


Supplementary figure 6: Correlation between FJX1 and drug IC50. FJX1 had significantly positive correlation with IC50 in LGK974, BMS-754807, Crizotinib,AZD5991, Vorinostat and ML_323.The value of “r” and “p” were shown.
